# An integrative genetic and transcriptomic study reveals a causal link and candidate biomarkers between tuberculosis and asthma

**DOI:** 10.3389/fgene.2026.1769766

**Published:** 2026-05-19

**Authors:** Yingxia Liu, Kun Li, Feifeng Guan, Yuanzhen Zhang, Ping Li, Min Yang, Huixing Tan, Caifen Chen, Lan Guo, Shumei Liu, Ming Shi, Jianhua Wang, Hancheng Liang

**Affiliations:** 1 Dongguan Sixth People’s Hospital, Dongguan, China; 2 Shenzhen University General Hospital, Shenzhen, China; 3 Guangdong Provincial Key Laboratory of Medical Immunology and Molecular Diagnostics, Guangdong Medical University, Dongguan, China

**Keywords:** asthma, comorbidity research, Mendelian randomization analysis, tuberculosis, WGCNA

## Abstract

**Introduction:**

Tuberculosis, caused by *Mycobacterium tuberculosis*, mainly affects the lungs, while asthma is a common chronic respiratory condition often linked with other health issues. Research on the connection between these two diseases is scarce, and their relationship needs more study.

**Methods:**

We analyzed data from the Global Burden of Disease study to compare the impact of tuberculosis and asthma worldwide from 2013 to 2023. We used two-sample Mendelian randomization to explore the causal link between tuberculosis, asthma, and lung function. Transcriptomic data from active tuberculosis and asthma patients were obtained from the Gene Expression Omnibus database for differential expression, gene co-expression network, and functional enrichment analyses. The expression patterns of the identified candidate genes were validated using quantitative PCR in an independent clinical cohort of 40 tuberculosis patients and 40 healthy controls.

**Results:**

The global disease burden analysis shows that tuberculosis has a greater impact than asthma. Mendelian randomization indicates that pulmonary tuberculosis is a risk factor for asthma (Odds ratio = 1.58, 95% Confidence interval: 1.08–2.31, p = 0.018 for ebi-a-GCST90086044; Odds ratio = 1.74, 95% Confidence interval: 1.43–2.12, p < 0.001 for ebi-a-GCST90086047) and adversely affects lung function, including forced vital capacity and forced expiratory volume at one second. Transcriptome analysis reveals immune pathway activation and cellular function suppression in both diseases. Using weighted gene co-expression network analysis, five comorbidity genes: *CRLF2*, *ETV5*, *LRRC1*, *OR10H1*, and *SENP2*, were identified. These genes show significant expression changes in tuberculosis patients and demonstrate high discriminatory potential in the discovery cohort, with an area under the curve of up to 1.0, supporting their further investigation as candidate biomarkers. Clinical validation confirmed these expression patterns.

**Conclusion:**

Tuberculosis is identified as a causal risk factor for asthma based on genetic evidence from Mendelian randomization. The five key genes, including *CRLF2* and *ETV5*, represent promising candidate biomarkers, providing new insights into their comorbidity.

## 1 Introduction

Tuberculosis (TB) is a persistent infectious disease caused by the bacterium *Mycobacterium tuberculosis* (*M.tb*) and is primarily transmitted via the respiratory tract. It represents a significant threat to human health and constitutes one of the major global public health challenges (WHO, 2024). Despite efforts to mitigate its impact, TB continues to be a substantial worldwide public health issue. The World Health Organization (WHO) has established the strategic objective of “End TB” by the year 2035. However, the efforts to prevent and control TB are hindered by persistent challenges in diagnosis, treatment, and prevention (John). *M.tb* is highly contagious, facilitating its spread within populations, and can inflict long-term damage on patients, significantly impairing their quality of life. In numerous low- and middle-income countries, *M.tb* remains a leading cause of morbidity and mortality. The disease is often exacerbated by factors such as HIV co-infection, poverty, and overcrowding (Dheda et al., 2016; Grüninger and Jost, 1995).

In contrast, asthma (AS) is a prevalent chronic respiratory condition characterized by a history of respiratory symptoms, including wheezing, shortness of breath, chest tightness, and coughing, which vary in frequency and intensity, alongside variable expiratory airflow limitation (Bousquet and Humbert, 2015). The impact of AS is evident not only in its effects on individuals of all ages globally but also in its frequent association with various comorbidities, including allergic rhinitis, gastroesophageal reflux disease, obstructive sleep apnea, and anxiety disorders (Althoff et al., 2021; Ye et al., 2021). These comorbidities substantially impair quality of life. Patients with AS are often at an elevated risk of comorbid infectious diseases, a health threat that is largely underestimated. Patients with AS are often at an elevated risk of comorbid infectious diseases, a health threat that is largely underestimated. Research indicates that immune function in individuals with AS may be compromised, resulting in increased susceptibility to viral infections (Juhn, 2017). Moreover, chronic allergic AS may lead to T cell dysfunction, impairing the body’s ability to eliminate viruses and thereby exacerbating health risks for AS patients (Ahn et al., 2023). *M.tb*, as a chronic infection with profound and lasting effects on the pulmonary immune landscape, may also predispose individuals to subsequent respiratory conditions such as AS.

A multicenter study conducted in the Nordic-Baltic region revealed that individuals with a history of TB exhibited a higher likelihood of developing AS and its associated symptoms, possibly reflecting TB’s long-term effects on the airways and lungs, as well as the involvement of inflammatory responses (Gyawali et al., 2023). But the relationship between TB and AS, and whether there are related mechanisms and target sites, remains inconclusive. Given the limited and inconclusive evidence on the temporal relationship between these two diseases, this study focuses on investigating whether TB serves as a causal risk factor for AS. In this study, we contextualized the comparative public health burden of the two diseases by utilizing data from the Global Burden of Disease (GBD) study, examined the causal relationship between TB and AS by employing Mendelian randomization (MR) techniques. Additionally, we investigated the impact of these diseases on lung function. Through the application of bioinformatics tools, we identified key genes between TB and AS. These findings were further validated using clinical samples.

## 2 Methods

### 2.1 GBD database and analysis

This study utilized data on AS and TB patients worldwide from 2013 to 2023, obtained from the GBD database. The data was used to quantify the impact of these diseases in terms of disability-adjusted life years (DALYs). The dataset comprises case numbers and DALYs, categorized by sex and age, and was accessed via the Global Health Data Exchange (GHDx) query tool (http://ghdx.healthdata.org/gbd-results-tool).

### 2.2 Two-sample mendelian randomization

**FIGURE 1:**
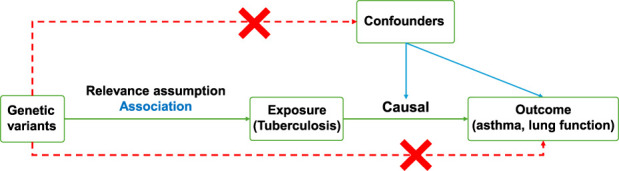
Schematic showing how MR was used to evaluate a causal association between TB traits with AS and lung function in this study.

To explore the causal relationship between TB and AS, we conducted an MR analysis. Figure 1 shows a schematic diagram of the study design. Data for pulmonary tuberculosis (ebi-a-GCST90018892, ebi-a-GCST90018672), asthma (ebi-a-GCST90086044, ebi-a-GCST90086045, ebi-a-GCST90086047), and lung function phenotypes (ebi-a-GCST007429, ebi-a-GCST007431, ebi-a-GCST90025978, ebi-a-GCST90029026) were sourced from genome-wide association studies (GWAS). Two-sample MR was conducted using the TwoSampleMR R package (version 0.5.6). The analysis employed inverse variance weighting (IVW), MR Egger, and weighted median models. The criteria for the models were established as follows: (1) the instrumental variables must be significantly associated with the exposure (p < 5e-8), and (2) the instrumental variables must not be associated with confounding factors. Subsequently, selected instrumental variables with unbalanced SNPs were also removed (clump = 10,000 kb, r^2^ < 0.001). For lung function outcomes, only one independent SNP (rs2385043) was available after clumping for the exposure GWAS used (ebi-a-GCST90018672), the causal estimate was derived using the Wald ratio method. The strength of this instrument was assessed using the F-statistic.

### 2.3 Mediation analysis

To assess whether the effect of TB on AS was mediated by lung function, we performed a two-step mediation analysis within the MR framework. The total effect of TB on AS was decomposed into a direct effect and an indirect effect mediated through lung function. The analysis was conducted using the ‘MRmediation’ R package, with standard errors estimated via the delta method.

### 2.4 Gene expression omnibus (GEO) data analysis

Transcriptional datasets of peripheral blood mononuclear cells (PBMCs) from TB and AS patients, specifically GSE62525 (TB n = 14, Healthy n = 14, South African participants) and GSE165934 (AS n = 10, Healthy n = 9, European participants), were retrieved from the GEO database. We acknowledge that gene expression patterns can vary across ethnicities, which represents a limitation when comparing signatures across datasets from different populations. It is important to note that these transcriptomic analyses are observational and identify associations. They are used to generate hypotheses about shared biology and are not intended as causal inference, which is addressed separately by our MR design. The datasets, initially unnormalized, underwent log2 transformation followed by normalization using the ‘normalize.quantiles’ function from the R package ‘preprocessCore’. Subsequently, probe identifiers were converted to gene symbols, and batch effects were mitigated using the ‘removeBatchEffect’ function from the R package ‘limma’. Differential gene expression analysis was performed to identify differentialy expressed genes (DEGs), utilizing the R package ‘limma’, applied the |FC| > 2 and adjusted p-value (FDR) < 0.05.

### 2.5 Construction of weighted gene co-expression network analysis (WGCNA)

We developed a co-expression network utilizing the WGCNA algorithm within the R package and RStudio environment (version 4.1.0). The parameter β serves as a soft-thresholding mechanism, enhancing strong gene correlations while excluding weaker ones. Upon determining the optimal β value, the network was constructed, and the corresponding dissimilarities were computed. Genes exhibiting similar expression profiles were grouped into gene modules. We conducted a correlation analysis between each module and disease phenotypes to identify modules significantly associated with TB and AS. Modules were primarily screened based on the highest absolute correlation coefficient with the disease trait. Modules were assigned arbitrary color names by the WGCNA algorithm (e.g., greenyellow, skyblue) following standard convention. These colors serve as labels for groups of co-expressed genes and do not carry biological significance. The statistical significance of the correlation for these top candidate modules was then validated, requiring an FDR <0.05.

### 2.6 Gene enrichment analysis

We conducted enrichment analysis of DEGs using the Database for Annotation, Visualization, and Integrated Discovery (DAVID) version 6.8, with annotations based on the Gene Ontology (GO) and Kyoto Encyclopedia of Genes and Genomes (KEGG) databases. An FDR <0.05 were established as the threshold for significant enrichment in both GO and KEGG analyses. Utilizing the R packages ‘Cluster Profiler’, GSEABase’, and RStudio (version 4.1.0), we performed Gene Set Enrichment Analysis (GSEA) on the intersecting genes of modules that demonstrated significant associations with TB and AS to identify functional pathways. The R package ‘enrichplot’ facilitated the generation of annotated visual representations of gene set enrichment maps. For all enrichment analyses (GO, KEGG, GSEA), an FDR <0.05 was considered statistically significant.

### 2.7 Collection of clinical samples

Peripheral blood samples were obtained from 40 TB patients and 40 healthy volunteers. The study protocols received approval from the Ethics Committee of Dongguan Sixth People’s Hospital (approval number: Z2023-002), and informed consent was secured from all participants. Diagnosis was established based on a combination of microbiological confirmation (positive sputum culture for *M.tb* and/or a positive Xpert MTB/RIF assay) and clinical-radiological criteria (consistent symptoms and suggestive chest imaging), in accordance with standard guidelines. PBMCs were isolated from all TB patients prior to the initiation of any anti-tuberculosis therapy. For both patients and controls, key exclusion criteria included HIV infection, other chronic lung diseases (COPD, bronchiectasis), autoimmune diseases, and recent use of immunosuppressive medications.

### 2.8 PBMC isolation

The collected blood was diluted in a 1:1 ratio with phosphate-buffered saline containing 10% sodium citrate and subjected to centrifugation at 160 × g for 20 min at room temperature. Following this initial centrifugation, the supernatant was discarded. The remaining sample underwent a second centrifugation at 800 × g for 20 min, allowing for the collection of PBMCs from the interface. The PBMCs were subsequently washed three times with RPMI medium and resuspended in complete RPMI medium.

### 2.9 RNA extraction

The isolated PBMCs were transferred into 200 µL of Trizol reagent and thoroughly mixed by pipetting. An RNA extraction solution, equivalent to one-fifth of the Trizol volume, was added, followed by vigorous shaking for 5 min and incubation at room temperature for an additional 5 min. The sample was then centrifuged at 10,000 × g at 4 °C for 15 min to collect the supernatant, which was subsequently mixed with an equal volume of isopropanol and incubated at room temperature for 10 min. The supernatant was discarded, and the precipitate was subsequently washed with pre-cooled 75% ethanol and subjected to centrifugation at 16,000 rpm at 4 °C for 5 min. Following the removal of the supernatant and air-drying of the RNA pellet, DEPC-treated water was added to fully dissolve the RNA. The concentration and purity of the RNA were then measured.

### 2.10 Quantitative PCR (qPCR)

Following RNA extraction, the RNA samples were reverse transcribed into complementary DNA utilizing the PrimerScriptTM RT Reagent Kit with gDNA Eraser (TaKaRa, Japan). The resulting cDNA was then analyzed through fluorescent quantitative PCR using the ABI 7900HT Real-Time PCR System (ROCHE, Switzerland). Relative quantification in the quantitative reverse transcription PCR experiments was performed employing the 2^−ΔΔCT^ method. Statistical differences between the TB patient group and the healthy control group for each candidate gene were assessed using an unpaired t-test. To control the false positive rate arising from multiple comparisons across the five candidate genes, the resulting p-values were adjusted using the Benjamini-Hochberg (BH) method to control the false discovery rate. We selected the human GAPDH gene as the housekeeping gene, the sequences of the qPCR primers are detailed in Table 1.

**TABLE 1:** qPCR primer sequences.

Gene	Forward Sequence(5′-3′)	Reverse Sequence(5′-3′)
CRLF2	AAG​CGA​CTG​GTC​AGA​GGT​GAC​A	GAG​GAG​AGA​CAC​CAT​CAG​AAG​G
ETV5	GTG​TTG​TGC​CTG​AGA​GAC​TGG​A	CGA​CCT​GTC​CAG​GCA​ATG​AAG​T
LRRC1	CAG​ACT​AAC​TCG​GAT​ACC​TGC​AG	CTG​GTT​GTC​AGA​TAG​CCA​CAG​AG
OR10H1	CCT​TCT​GCT​GAA​GGT​CGG​AAC​A	GGC​TTC​AGG​TAA​ATG​ACG​GAG​G
SENP2	CAG​AGA​CGA​TGG​TCG​GAA​TCA​G	CCT​CCT​GAG​TAA​GCC​ATT​GCT​TC
GAPDH	GTC​TCC​TCT​GAC​TTC​AAC​AGC​G	ACC​ACC​CTG​TTG​CTG​TAG​CCA​A

### 2.11 Data analysis

Each experiment was conducted a minimum of three times, and the results are displayed in bar graphs as the mean ± standard deviation. Statistical differences between the means were assessed using a t-test.

## 3 Result

### 3.1 The global burden of tuberculosis and asthma: a descriptive epidemiological context

We collected epidemiological data on global TB and AS patients from 2013 to 2023. Our analysis revealed that the global disease burden of TB surpassed that of AS in terms of both case numbers and DALYs (Supplementary Figures S1A,B). Males experienced a higher overall disease burden for both diseases than females, particularly among adults aged 30–59 years (Supplementary Figure S1C). The DALY contribution from TB in males exceeded that from TB in females and from AS in both sexes across all age groups (Supplementary Figure S1D). Annual trends in DALY proportions and mortality rates are presented descriptively (Supplementary Figures S1E,F). These data illustrate that TB and asthma are major chronic respiratory diseases with substantial, though distinct, global burdens, providing background for our subsequent investigation into potential causal or molecular links using MR and transcriptomic analyses.

### 3.2 Two-sample MR elucidates the causal effect of TB on AS and lung function

**FIGURE 2:**
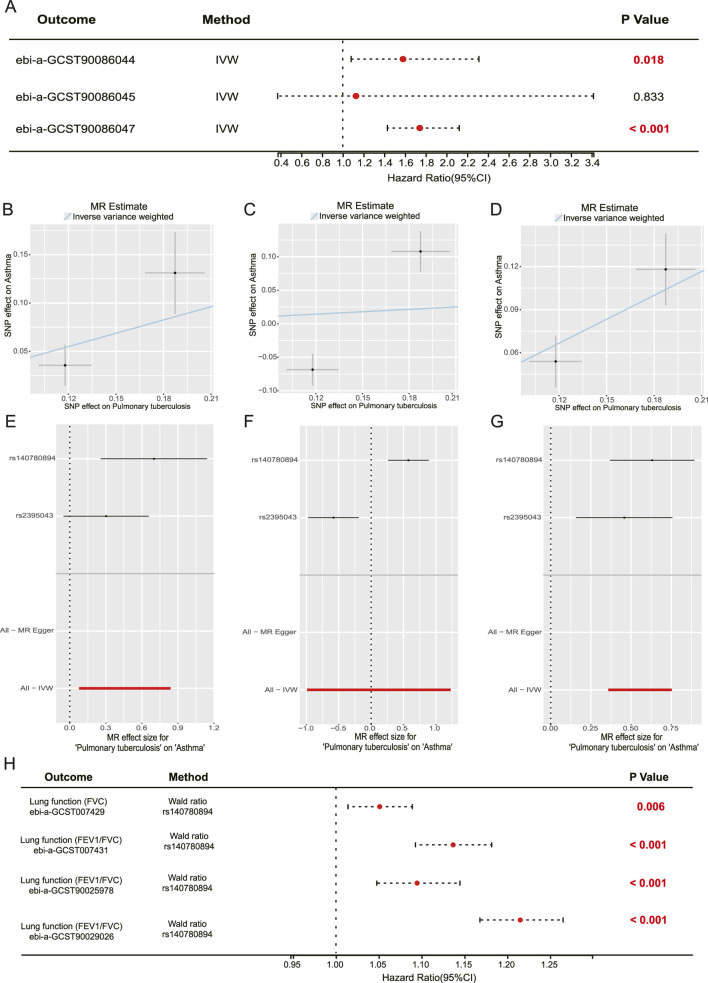
The MR analysis examines the causal relationships between TB, AS, and lung function. **(A)** Forest plot illustrates the causal effect estimates, with TB as the exposure variable and three AS GWAS datasets (ebi-a-GCST90086044, ebi-a-GCST90086045, ebi-a-GCST90086047) as the outcome variables. **(B–D)** Scatter plots corresponding to the MR analysis results for the three AS datasets mentioned in **(A)**. Each point on these plots represents an instrumental variable, specifically a single nucleotide polymorphism (SNP), with its position determined by its association with the exposure (TB) on the X-axis and with the outcome (AS) on the Y-axis. The trend lines indicate the direction of the causal effect as estimated by the MR-Egger and IVW methods. **(E–G)** The results of the leave-one-out sensitivity analysis, MR estimates are recalculated after sequentially removing one SNP at a time to assess the robustness of the findings. The proximity of all points to the overall estimate (represented by the horizontal line) suggests that the results are not unduly influenced by any single SNP. **(H)** Forest plot displaying the causal effect estimates with TB as the exposure and various lung function measures with single SNP (rs2385043) in Wald ratio, specifically FVC and the FEV1/FVC, as the outcomes.

In order to investigate the correlation and underlying mechanisms between TB and AS, we conducted a two-sample MR analysis. pulmonary tuberculosis (ebi-a-GCST90018892) was designated as the exposure variable, while AS served as the outcome variable. The analysis revealed a causal relationship between TB and an elevated risk of AS, as evidenced by the results from ebi-a-GCST90086044 (IVW: Odds Ratio [OR] = 1.581, 95% Confidence Interval [CI]: 1.081–2.313, p = 0.018) and ebi-a-GCST90086047 (IVW: OR = 1.742, 95% CI: 1.430–2.123, p < 0.001) (Figure 2A). Conversely, the results for ebi-a-GCST90086045 did not reach statistical significance (IVW: OR = 1.127, 95% CI: 0.372–3.417, p = 0.833) (Figures 2A–D). The robustness of these findings was further corroborated through leave-one-out cross-validation (Figures 2E–G). Additionally, we explored the causal relationship between TB and lung function using a similar two-sample MR approach, thereby enhancing our understanding of the association between TB and AS. Pulmonary tuberculosis (ebi-a-GCST90018672) was again used as the exposure, with lung function measures such as forced vital capacity (FVC) (ebi-a-GCST007429) and the forced expiratory volume in one second to forced vital capacity ratio (FEV1/FVC) (ebi-a-GCST007431, ebi-a-GCST90025978, ebi-a-GCST90029026) serving as outcome variables. Using the single strong SNP instrument (rs2385043, F-statistic = 98.7), the analysis demonstrated a causal association between TB and an elevated risk of reduced FVC, as indicated by a Wald ratio with an OR of 1.051, a 95% CI of 1.014–1.089, and a p-value of 0.0059. Furthermore, a causal relationship was identified between tuberculosis and an increased risk of reduced forced expiratory volume in FEV1/FVC, supported by data from ebi-a-GCST007431 (Wald ratio: OR = 1.254, 95% CI: 1.210–1.299, p < 0.001), ebi-a-GCST90025978 (Wald ratio: OR = 1.212, 95% CI: 1.165–1.262, p < 0.001), and ebi-a-GCST90029026 (Wald ratio: OR = 1.215, 95% CI: 1.168–1.265, p < 0.001) (Figure 2H). We found lung function does not mediate the relationship between TB and AS (Supplementary Figure S2). These MR analyses provide genetic evidence supporting a causal role of pulmonary tuberculosis in increasing the risk of AS and impairing lung function (Supplementary Tables S1-S5).

### 3.3 Observational transcriptomic analysis of TB and AS PBMC data reveals common and distinct pathway alterations

**FIGURE 3:**
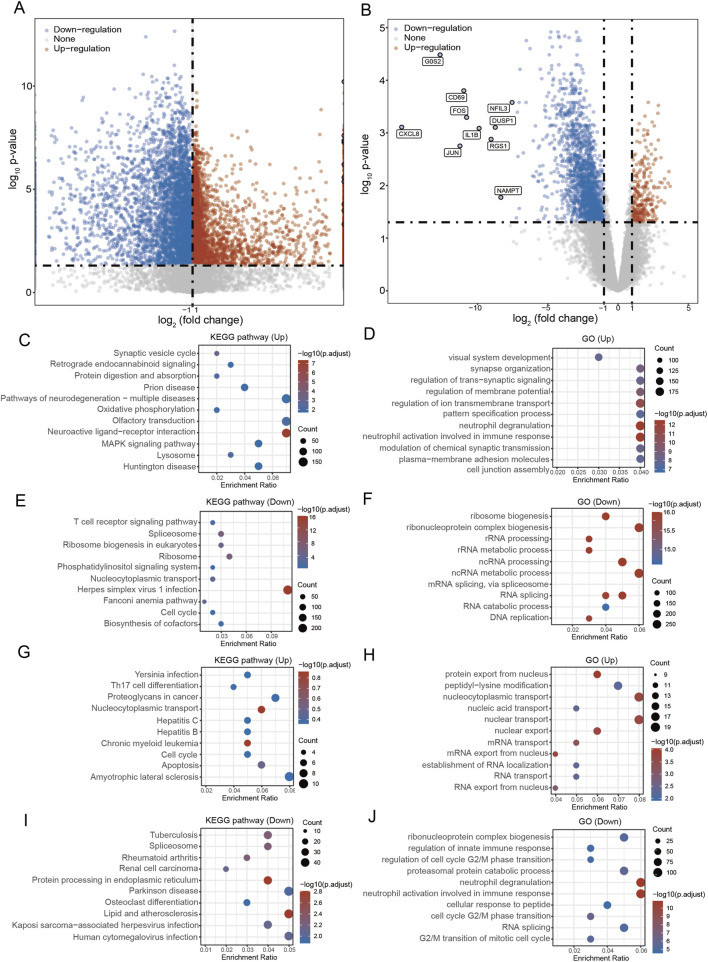
Differential expression and functional enrichment analysis of PBMCs in TB and AS patients. **(A)** Volcano plot of DEGs comparing the TB group with healthy controls. Red dots represent significantly upregulated genes, and blue dots represent significantly downregulated genes. **(B)** Volcano plot of DEGs comparing the AS group with healthy controls. **(C,D)** Bubble plots showing GO function and KEGG pathway enrichment analysis of upregulated genes in TB. The results indicate that these genes are significantly enriched in immune-inflammatory pathways. **(E,F)** Bubble plots showing GO function and KEGG pathway enrichment analysis of downregulated genes in TB. The results indicate that these genes are significantly enriched in basic cellular processes such as ribosome biogenesis, RNA splicing and metabolism, DNA replication, and the cell cycle. **(G,H)** Bubble plots showing GO function and KEGG pathway enrichment analysis of upregulated genes in AS. The results indicate that these genes are closely associated with Th17 cell differentiation, viral infection, and protein/mRNA transport. **(I,J)** Bubble plots showing GO function and KEGG pathway enrichment analysis of downregulated genes in AS. The results indicate that these genes are mainly involved in processes such as ribonucleoprotein complex assembly, RNA transport, and G2/M cell cycle transition.

To explore potential molecular associations between TB and AS, we performed comparative transcriptomic profiling of PBMCs from patients with these conditions. This analysis aimed to uncover molecular-level associations between the two diseases (Supplementary Figure S2). The transcriptomic profiling revealed notable alterations in gene expression for both TB and AS, Using the criteria of FDR <0.05 and |FC| > 2, we identified 9,194 and 2089 DEGs in the TB and AS dataset (Figures 3A,B; Supplementary Table S6). In the TB dataset, functional enrichment analysis of DEGs indicated that upregulated genes were significantly associated with immune-inflammatory pathways (Figures 3C,D). Conversely, downregulated genes were predominantly linked to essential cellular processes, including ribosome biogenesis, RNA splicing and metabolism, DNA replication, and the cell cycle (Figures 3E,F). In the AS dataset, functional analysis of DEGs revealed that upregulated genes were closely related to Th17 cell differentiation, viral infection, and protein/mRNA transport (Figures 3G,H). In contrast, downregulated genes were primarily involved in ribonucleoprotein complex assembly, RNA transport, and the G2/M transition of the cell cycle (Figures 3I,J). These findings indicate that both diseases demonstrate active immune responses, accompanied by a broad suppression of fundamental cellular functions.

### 3.4 WGCNA analysis of TB and AS datasets

**FIGURE 4:**
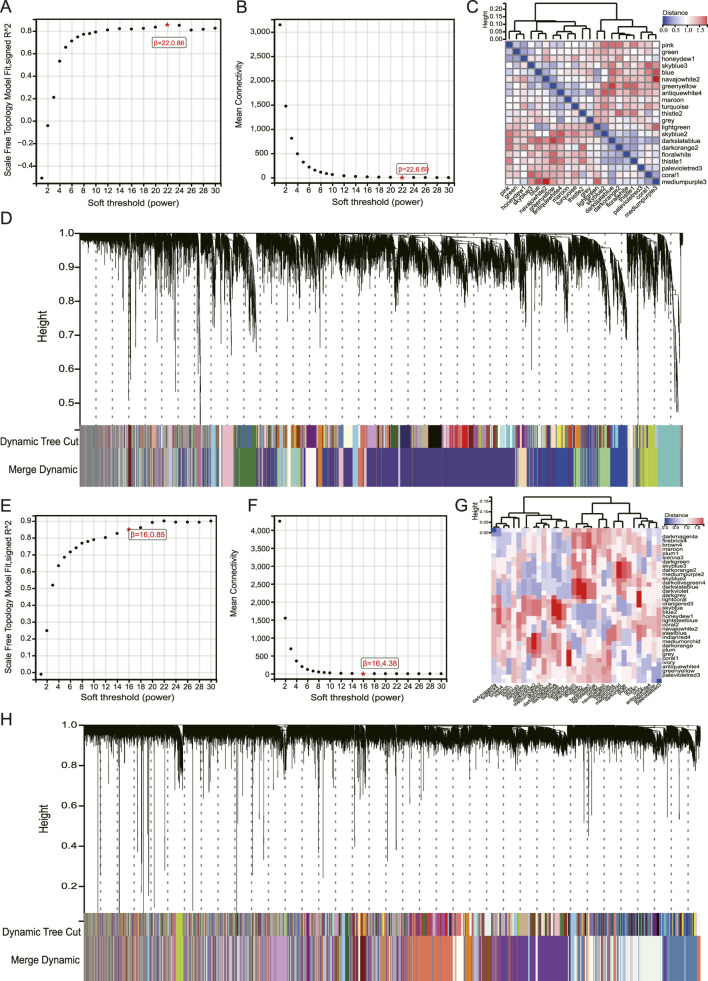
WGCNA of TB and AS Transcriptomic Datasets. **(A,B)** Soft-threshold selection for the TB dataset. **(A)** Shows the scale-free topology fit index (signed R^2^) for different soft-threshold powers. Power = 22 (R^2^ = 0.86) was chosen to approximate a scale-free network. **(B)** Shows the average connectivity for different soft-threshold powers; the average connectivity at power = 22 is 6.69. **(C)** Hierarchical clustering dendrogram of genes in the TB dataset based on gene expression similarity, with different colors representing different co-expression gene modules identified by the dynamic tree cut algorithm. **(D)** Dynamic dendrogram of merged modules in the TB dataset, showing the final merged gene modules. **(E)** Scale-free topology fit index plot for the AS dataset, with power = 16 (R^2^ = 0.85) chosen as the soft-threshold. **(F)** Average connectivity plot for the AS dataset, with average connectivity at power = 16 being 4.38. **(G)** Hierarchical clustering dendrogram of genes in the AS dataset and module colors identified by dynamic tree cutting. **(H)** Dynamic dendrogram of merged modules in the AS dataset.

To systematically investigate the co-regulatory patterns of gene expression in TB and AS, we employed WGCNA on these diseases. The TB (Figures 4A–D) and AS (Figures 4E–H) datasets were processed using soft thresholding powers of 22 and 16, respectively, to ensure that the resultant networks exhibited scale-free topology characteristics, as indicated by a scale-free topology fit index R^2^ greater than 0.85, while maintaining suitable average connectivity (Figures 4A,B,E,F). Hierarchical clustering based on gene expression similarity, in conjunction with a dynamic tree cut algorithm, facilitated the identification of multiple co-expression gene modules, each distinguished by unique colors (Figures 4C,D,G,H). The dendrograms of these modules revealed that genes within each module exhibited strong co-expression, whereas the modules themselves were distinctly separated, underscoring the robustness of the network construction and the validity of the module delineation. These identified gene modules serve as a foundational basis for subsequent analyses aimed at elucidating associations with key clinical traits or pathological states.

**FIGURE 5:**
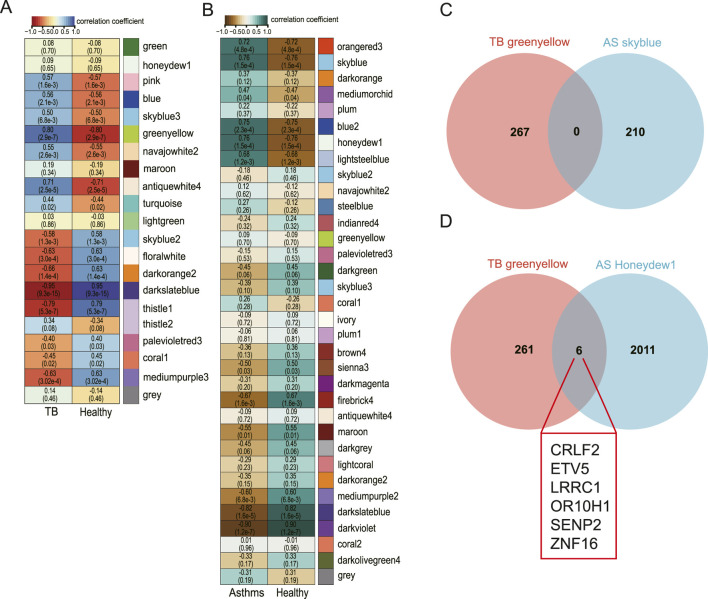
Analysis of co-expression modules associated with disease traits and screening of comorbidity genes. **(A)** Heatmap showing the association of gene modules with disease status (TB vs. Healthy) in the TB dataset. The color intensity represents the correlation coefficient, and the value in parentheses is the corresponding P-value. Among them, the darkslateblue module is strongly negatively correlated with TB (r = −0.95), while the greenyellow module shows a significant positive correlation (r = 0.80). **(B)** Heatmap showing the association of gene modules with disease status (Asthma vs. Healthy) in the AS dataset. The darkviolet module is significantly negatively correlated with AS, whereas the skyblue and honeydew1 modules are significantly positively correlated with AS. **(C,D)** Venn diagrams showing the gene overlap between TB positively correlated modules (greenyellow) and AS positively correlated modules (skyblue, honeydew1). The results show that there are six common genes between the greenyellow and honeydew1 modules (CRLF2, ETV5, LRRC1, OR10H1, SENP2, ZNF16).

To investigate the biological associations between co-expressed gene modules and disease states, we performed a module-trait association analysis. Our findings revealed that several gene modules exhibited significant correlations with TB and AS (Figures 5A,B. Notably, the darkslateblue module demonstrated a strong negative correlation with TB (r = −0.95, p = 9.3e-15), whereas the greenyellow module was significantly positively correlated with TB (r = 0.80, p = 2.9e-7). In AS, the darkviolet module was significantly negatively correlated (r = −0.90, p = 1.2e-7), while the skyblue and honeydew1 modules showed significant positive correlations (r = 0.76, p = 1.5e-4). We further examined genes from the TB greenyellow module and the AS skyblue and honeydew1 modules, discovering no overlap between the TB greenyellow module and AS skyblue genes. However, six genes were common between the TB greenyellow module and the AS honeydew1 module (*CRLF2*, *ETV5*, *LRRC1*, *OR10H1*, *SENP2*, *ZNF16*) (Figures 5C,D). These results elucidate the disease-specific associations of co-expression network modules and offer crucial insights into the molecular regulatory mechanisms underlying TB and AS.

### 3.5 Validation of the diagnostic value of comorbid genes in TB and AS

**FIGURE 6:**
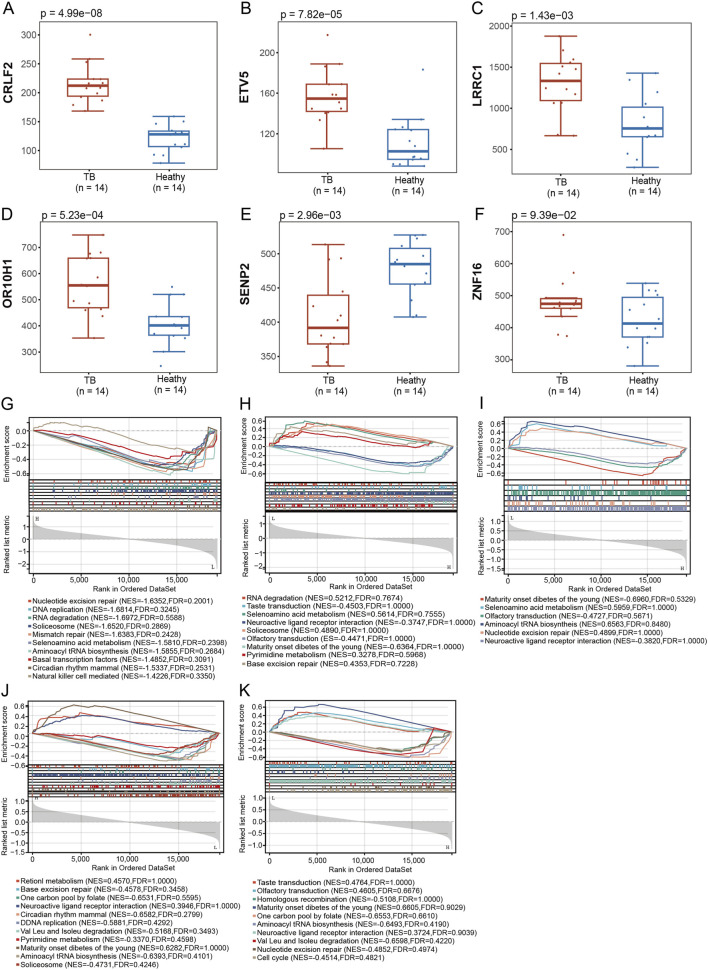
Verification of comorbidity gene expression in TB and functional pathway analysis. Box plots show the expression levels of six comorbidity genes in the peripheral blood of TB patients (n = 14) and healthy controls (n = 14). Compared with the healthy controls, TB patients showed significantly upregulated expression of **(A)** CRLF2, **(B)** ETV5, **(C)** LRRC1, and **(D)** OR10H1, while **(E)** SENP2 expression was significantly downregulated. **(F)** ZNF16 expression showed no significant difference. The GSEA results depict the signaling pathways most correlated with the expression profiles of **(G)** CRLF2, **(H)** ETV5, **(I)** LRRC1, **(J)** OR10H1, and **(K)** SENP2. The results indicate that in the TB group, these genes are closely associated with significant suppression of genetic information processing pathways such as nucleotide excision repair, DNA replication, mismatch repair, and RNA degradation.

To substantiate the findings of the co-expression network analysis, we assessed the expression levels of the shared genes within the module in TB datasets. The analysis revealed that, in the TB PBMC dataset, TB patients demonstrated a significant upregulation of *CRLF2* (Figure 6A, p = 4.99e-08), *ETV5* (Figure 6B, p = 7.82e-05), *LRRC1* (Figure 6C, p = 1.43e-03), and *OR10H1* (Figure 6D, p = 5.23e-04; Supplementary Tables S7, S8; Supplementary Figure S3) compared to healthy controls, while *SENP2* was significantly downregulated (Figure 6E, p = 2.96e-03). These findings further corroborate the potential involvement of these genes in the pathogenesis of TB. Conversely, *ZNF16* (Figure 6F, p = 0.093) did not exhibit a statistically significant difference. To further elucidate their biological significance, we conducted GSEA. The results indicated that, within the TB cohort, *CRLF2* (Figure 6G), *ETV5* (Figure 6H), *LRRC1* (Figure 6I), *OR10H1* (Figure 6J), and *SENP2* (Figure 6K) were significantly inhibited in pathways associated with genetic information processing, including nucleotide excision repair, DNA replication, mismatch repair, and RNA degradation. This suggests that *M.tb* infection may have a broad impact on the fundamental maintenance functions of host cells. The collective findings at the gene expression and pathway levels suggest that TB induces extensive and significant suppression of fundamental host cell functions, particularly affecting DNA repair and replication pathways. Similarly, AS DEGs exhibited comparable pathway disruptions (Figure 3).

**FIGURE 7:**
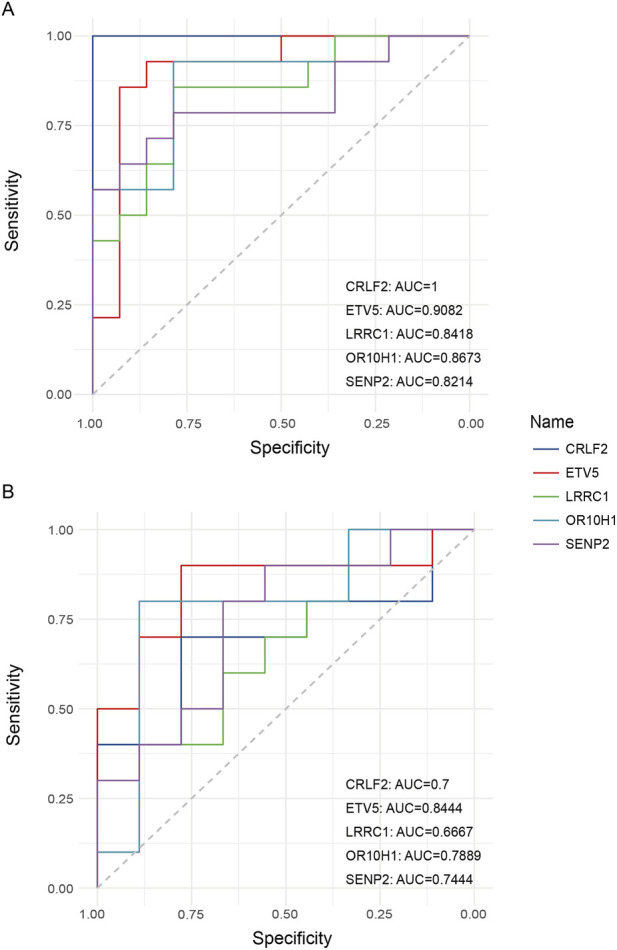
Evaluation of the discriminatory capacity in the discovery dataset of key comorbidity genes for TB and AS. **(A)** ROC curves showing the ability of 5 key genes (CRLF2, ETV5, LRRC1, OR10H1, SENP2) to distinguish TB patients from healthy controls in the TB dataset. All genes exhibited good diagnostic value, with CRLF2 achieving an AUC of 1, AUC values from the discovery dataset should be interpreted as preliminary; independent validation is required. **(B)** ROC curves showing the ability of the same 5 key genes to distinguish AS patients from healthy controls in the AS dataset. All genes showed certain diagnostic potential, with ETV5 having the highest AUC (0.8444). AUC values from the discovery dataset should be interpreted as preliminary; independent validation in a separate cohort is required.

To assess the diagnostic efficacy of these five genes, we performed diagnostic analyses on TB and AS datasets. In the TB cohort, all five genes demonstrated robust diagnostic performance, with *CRLF2* exhibiting the highest discriminative capacity, achieving a receiver operating characteristic (ROC) curve area under the curve (AUC) of 1, indicative of exceptionally high sensitivity and specificity. Additionally, *ETV5* (AUC = 0.9082), *LRRC1* (AUC = 0.8418), *OR10H1* (AUC = 0.8673), and *SENP2* (AUC = 0.8214) also demonstrated significant diagnostic value (Figure 7A). In the AS cohort, *ETV5* maintained high sensitivity and specificity with a ROC curve AUC of 0.8444, while the AUC values for the other genes were as follows: *CRLF2*: AUC = 0.7, *LRRC1*: AUC = 0.6667, *OR10H1*: AUC = 0.7889, and *SENP2*: AUC = 0.7444 (Figure 7B). These findings suggest that the identified key genes, particularly *CRLF2* and *ETV5*, represent promising candidate biomarkers warranting further investigation for TB and AS.

### 3.6 Validation of comorbidity genes in a clinical sample

**FIGURE 8:**
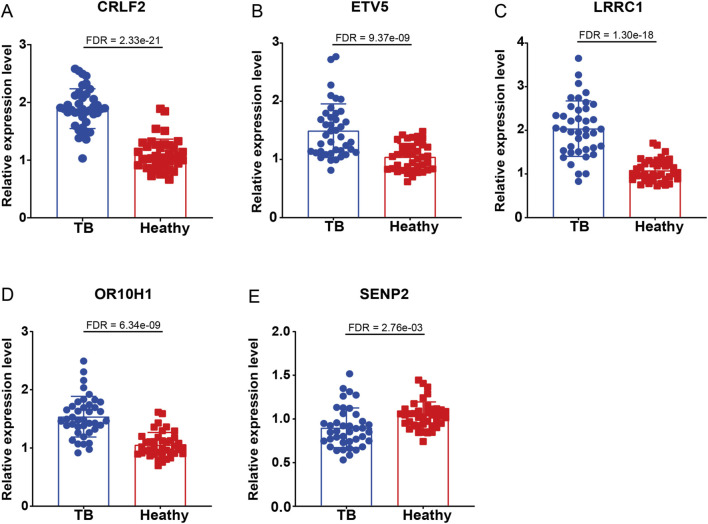
qPCR validation of comorbidity genes in clinical samples from TB patients. The relative mRNA expression levels of key comorbidity genes in PBMCs from 40 TB patients and 40 healthy volunteers were detected by qPCR. The results showed that, compared with the healthy control group, the expression of **(A)** CRLF2, **(B)** LRRC1, **(C)** ETV5, and **(D)** OR10H1 were significantly upregulated in TB patients, while the expression of **(E)** SENP2 were significantly downregulated. P-values were adjusted for multiple testing across the five genes using the Benjamini-Hochberg false discovery rate (FDR) method.

To further substantiate the selection of comorbidity genes, blood samples were obtained from patients with TB and from healthy volunteers (Supplementary Table S9). PBMCs were extracted from these samples, and the expression levels of the target genes were quantified using qPCR. The analysis revealed that, in comparison to the healthy control group, the genes *CRLF2* (FDR = 2.33e-21), *ETV5* (FDR = 9.37e-09), *LRRC1* (FDR = 1.30e-18), and *OR10H1* (FDR = 6.34e-09) was significantly upregulated in TB patients, while *SENP2* expression was significantly downregulated (FDR = 2.76e-03) (Figure 8). These findings align with prior transcriptomic data and module-trait association analyses, thereby reinforcing the potential critical role of these genes in TB pathogenesis and supporting their utility as reliable molecular markers.

## 4 Discussion

TB remains a highly prevalent infectious disease, with significant implications for respiratory health. A substantial proportion of individuals with pulmonary TB exhibit varying levels of respiratory dysfunction, which is a major contributor to TB-related mortality (Kugelberg, 2016; Nightingale et al., 2022). The effect of TB on pulmonary function constitutes a critical public health concern. Pulmonary TB not only induces structural damage to the lungs but also contributes to a progressive decline in lung function over time. Evidence from a systematic review and meta-analysis indicates that individuals with a history of TB are at a markedly increased risk of developing chronic obstructive pulmonary disease (COPD), with an odds ratio of 2.59. This suggests that TB patients are 2.59 times more likely to suffer from compromised lung function compared to the general population (Sarkar et al., 2017). Furthermore, research focusing on pediatric patients who have completed TB treatment reveals that these children exhibit significantly impaired lung function and reduced health-related quality of life relative to their peers without a TB history. This underscores the necessity of addressing the long-term consequences of TB in the pediatric demographic (Nkereuwem et al., 2023).

AS is a prevalent chronic respiratory condition whose detrimental effects extend beyond the respiratory system, potentially impacting overall health. The interplay between TB and AS represents a complex and multifaceted area of research. The significant impact of TB on the immune system may affect the onset and progression of AS. *M.tb* infection can induce both local and systemic immune and inflammatory alterations, potentially influencing the development of other respiratory disorders. In GBD analysis, the concurrent dips in burden metrics for both diseases post-2019 likely reflect the pervasive impact of the COVID-19 pandemic on healthcare systems and disease reporting, rather than a specific biological interaction (Supplementary Figure S1). Using MR, our findings indicate that TB constitutes a risk factor for AS (IVW: OR = 1.581, p = 0.018; IVW: OR = 1.742, p < 0.001; Figure 2). Additionally, the observed causal relationship between TB and AS may involve shared genetic architecture or pleiotropic effects of genetic variants, a possibility supported by the use of genetic instruments in MR. The G-308A polymorphism of the tumor necrosis factor-alpha gene, for instance, has been found to be significantly correlated with AS occurrence, although its association with TB was not significant in previous studies (Kumar et al., 2008). Additionally, the association between inhaled corticosteroids (ICS) and TB risk merits attention. Evidence indicates that ICS usage is linked to an elevated risk of TB development, particularly among patients diagnosed with COPD (Kumar et al., 2008). This finding indicates that caution should be exercised when administering ICS to patients with AS, particularly those with a history of TB (Lee et al., 2019; Ni et al., 2014).

*M.tb* infection can induce permanent alterations in lung anatomy, affecting airflow and resulting in dyspnea and other associated symptoms. In elderly individuals, the impact of TB may be more pronounced due to age-related decline in pulmonary immune defenses and changes in the pulmonary mucosal environment, which may increase susceptibility to *M.tb* infection. The alveolar surface liquid in older adults exhibits heightened oxidative and inflammatory responses upon interaction with *M.tb*, potentially diminishing the ability to control the infection (Murray and Chotirmall, 2015). Consequently, pulmonary function impairment following *M.tb* infection may be more severe in older adults. Our findings indicate that TB not only has a causal relationship with AS but also significantly impacts lung function (Figure 2H), Furthermore, the MR analysis for lung function relied on a single genetic instrument, which precluded the application of pleiotropy-robust sensitivity analyses applicable to multi-SNP estimates. However, lung function does not mediate the relationship between TB and AS (Supplementary Figure S3). Therefore, the association between TB and AS warrants further comprehensive investigation.

Having established a potential causal link, we next sought to identify shared gene expression patterns that might inform the underlying biology of this comorbidity. In this study, we investigated the genetic association between TB and AS utilizing data from the GEO. DEGs in PBMCs from patients with TB and AS were analyzed using WGCNA, leading to the identification of the most highly correlated comorbidity genes (Figures 3–6). Among these, five comorbidity genes—*CRLF2*, *ETV5*, *LRRC1*, *OR10H1*, and *SENP2*—exhibited significant differences in expression when compared to healthy controls within the TB dataset (Figures 6A–F). The expression patterns in asthma were derived from the public transcriptomic dataset (GSE165934), and further experimental validation in AS patients, while valuable, was beyond the scope of this study and represents an important direction for future research. Subsequent in-depth analysis of these genes revealed their substantial diagnostic value for TB (Figure 7), which was further corroborated through validation with clinical samples (Figure 8). It should be noted that our assessment of diagnostic performance was based primarily on the candidate genes themselves, without constructing multivariable models incorporating traditional risk factors. Although the balanced distribution of age and sex in the validation cohort mitigates concerns about confounding to some extent, future studies in larger populations are needed to evaluate the incremental value of these markers beyond clinical variables.

*CRLF2* encodes a cytokine receptor that forms a complex with TSLPR to bind thymic stromal lymphopoietin (*TSLP*), an “alarmin” produced by epithelial cells in response to damage, inflammation, or pathogen invasion (Tian et al., 2023). Upon *TSLP* binding, *CRLF2* activates downstream *JAK/STAT* signaling pathways, influencing the development and function of immune cells (Sia et al., 2020). This signaling axis promotes dendritic cell maturation and enhances Th1-type cytokine production, such as *IL-12*, which is critical for effective host defense against intracellular pathogens like *M. tb* (Mullighan et al., 2009). In our study, *CRLF2* was significantly upregulated in TB patients (Figures 6A, 8A), suggesting a potential role in the anti-mycobacterial immune response. In asthma, while the role of *CRLF2* is less defined, *TSLP* signaling has been implicated in type 2 inflammation, and pathogen-elicited immune responses may modulate disease progression. Thus, *CRLF2* may represent a shared molecular node in the immune pathophysiology of both diseases. *ETV5* is a transcription factor involved in the maintenance of alveolar epithelial cells and immune regulation (Zeng et al., 2025; Shi et al., 2024). It is essential for preserving alveolar type II cell identity, and its deficiency impairs lung repair following injury (Zhang et al., 2017). Given that alveolar type II cells are integral to pulmonary immune defense, alterations in this cell population may influence *M. tb* colonization and transmission. Additionally, *ETV5* plays a critical role in Th17 cell differentiation by recruiting histone-modifying enzymes, and it is essential for Th17 function in allergic airway inflammation, as evidenced by reduced airway inflammation and *IL-17A/F* production in *ETV5*-deficient T cells (Zhang et al., 2025; Pham et al., 2014). Our finding that *ETV5* is upregulated in TB patients (Figures 6B, 8C) suggests it may contribute to pulmonary inflammatory responses in both infectious and allergic contexts. *LRRC1* is a potential regulator of cell polarity that may modulate pathogen invasion and intracellular survival by influencing cytoskeletal remodeling (Song et al., 2018). *M. tb* employs various mechanisms to evade host immune clearance, and *LRRC1* could play a role in these processes (Fu et al., 2020). Its expression changes in TB patients (Figures 6C, 8B) warrant further investigation into its function in host-pathogen interactions. *OR10H1* is an olfactory receptor that has been associated with host defense mechanisms mediated by circulating neutrophils, which capture and kill pathogens through the formation of neutrophil extracellular traps (*NETs)* (Brinkmann et al., 2004; Donkel et al., 2021), While its canonical role is in olfactory signaling, ectopic expression of olfactory receptors in non-olfactory tissues, including immune cells, has been increasingly recognized (Weber et al., 2018). Our observation of *OR10H1* upregulation in TB patients (Figures 6D, 8D) suggests a possible link to neutrophil-mediated immunity. SENP2 is a deSUMOylating enzyme that modulates antiviral innate immunity by regulating IRF3 ubiquitination and degradation (Ran et al., 2011). It also suppresses *NF-κB* activation via NEMO deSUMOylation and influences macrophage polarization, specifically inhibiting M1 polarization and inflammatory mediator production (Zheng et al., 2025). *SENP2* expression was downregulated in TB patients (Figures 6E, 8E), suggesting that *M. tb* may modulate host immune responses by altering deSUMOylation dynamics. This finding aligns with the broad suppression of fundamental cellular processes observed in our transcriptomic analysis (Figure 3).

In conclusion, our MR analysis suggests a causal relationship from TB to AS. Independently, our transcriptomic co-expression analysis identified genes such as *CRLF2* and *ETV5* as being associated with both conditions. We hypothesize that these genes may point to shared biological pathways relevant to the comorbidity, a possibility that warrants future functional investigation. By synthesizing epidemiological, genetic, and bioinformatics methodologies, and utilizing GBD data, we elucidated the macro-level associations of disease burden. Employing two-sample MR analysis, we established causal relationships from TB to asthma and lung function. Additionally, we identified core gene modules and candidate genes (*CRLF2*, *ETV5*, *LRRC1*, *OR10H1*, *SENP2*) associated with both diseases through transcriptomic analysis and WGCNA. The expression alterations of these genes in TB patients were validated using qPCR experiments on independent clinical samples, affirming their reliability. This study not only provides genetic evidence that TB is a risk factor for asthma but also identifies a set of key genes related to comorbidity, offering novel insights into the pathophysiological mechanisms underlying these two significant chronic lung diseases and candidate diagnostic markers for their auxiliary diagnosis. Nonetheless, this study is subject to certain limitations. First, MR analysis indicates a causal relationship, the precise biological pathways involved, and the specific roles of comorbid genes necessitate further functional validation through molecular biology experiments. Subsequently, several limitations should be considered when interpreting our MR findings: **(a)** the primary MR analysis combined an East Asian exposure GWAS with European outcome GWAS, which may introduce bias due to ancestral differences in genetic architecture, although consistent directional effects across multiple independent asthma outcomes support robustness; **(b)** the MR analysis for lung function relied on a single genetic instrument (rs2385043), which precluded the application of pleiotropy-robust sensitivity analyses; and **(c)** while MR provides genetic evidence for a causal relationship, the specific biological mechanisms underlying this association require further functional investigation. Third, the clinical validation cohort in this study comprised only 40 cases, which is inadequate for a comprehensive verification of the clinical applicability of comorbidity genes. The diagnostic performance reported here should be considered exploratory; definitive evaluation of clinical utility requires prospective studies in large, well-characterized populations. Future research endeavors should aim to enhance the robustness of these findings by increasing the number of clinical samples. Fourth, although our identified candidate genes (e.g., *CRLF2*) showed exceptional discriminatory capacity (AUC up to 1.0) in the transcriptomic discovery cohort, these estimates likely represent an upper bound of performance due to the risk of overfitting in small sample sizes. The absence of an internal cross-validation partition is a limitation. Fifth, our study involved multiple populations with different genetic backgrounds and environmental exposures: the TB exposure GWAS was from East Asian, the asthma outcome GWAS and asthma transcriptome from European, and the TB transcriptome from South African participants. Factors such as air quality, BCG vaccination policies, and genetic ancestry may influence gene expression patterns and the generalizability of our findings. However, the candidate genes identified (such as ETV5) are evolutionarily conserved transcription factors with well-documented roles in immune regulation across mammals, which may partly mitigate concerns about population-specific effects. Nevertheless, future studies using ancestrally matched, multi population cohorts are needed to confirm the universality of these candidate markers. The subsequent validation of gene expression changes in a fully independent clinical cohort (n = 80) strengthens the biological plausibility of these candidates. Their definitive diagnostic utility must be established through future prospective studies in large, independent populations. Additionally, although this study identifies an association between lung function and both diseases, the mediating effect is not significant. The specific mechanistic pathway through which tuberculosis contributes to the development of asthma requires further investigation.

## Data Availability

The original contributions presented in the study are included in the article/Supplementary Material, further inquiries can be directed to the corresponding authors.

## Supplementary material

SUPPLEMENTARY FIGURE S1 Global burden of tuberculosis (TB) and asthma (AS) from 2013 to 2023. **(A)** Bar chart showing the number of TB and AS cases. **(B)** Bar chart showing DALY rates for TB and AS. **(C)** Distribution of DALYs by gender across age groups (15–59 years). This panel illustrates the age-specific patterns for each disease by sex. **(D)** Stacked bar chart showing the cumulative contribution of each age group to total DALYs, stratified by sex and disease. Each segment represents one age group; the total bar height represents the sum across all age groups. **(E)** Annual trends in the proportion of DALYs attributable to TB and AS relative to all-cause DALYs. **(F)** Annual trends in the proportion of deaths attributable to TB and AS relative to all-cause mortality.

SUPPLEMENTARY FIGURE S2 GEO Dataset Analysis.

SUPPLEMENTARY FIGURE S3 Mediation MR Analysis of TB, AS, and Lung Function.

SUPPLEMENTARY FIGURE S4 Correlation of OR10H1 expression with immune cell subsets.
