# Supplementary figures and images for: An integrative genetic and transcriptomic study reveals a causal link and candidate biomarkers between tuberculosis and asthma
Source: Front Genet. 2026 May 19;17:1769766. doi: 10.3389/fgene.2026.1769766 (PMC13225779; doi:10.3389/fgene.2026.1769766)

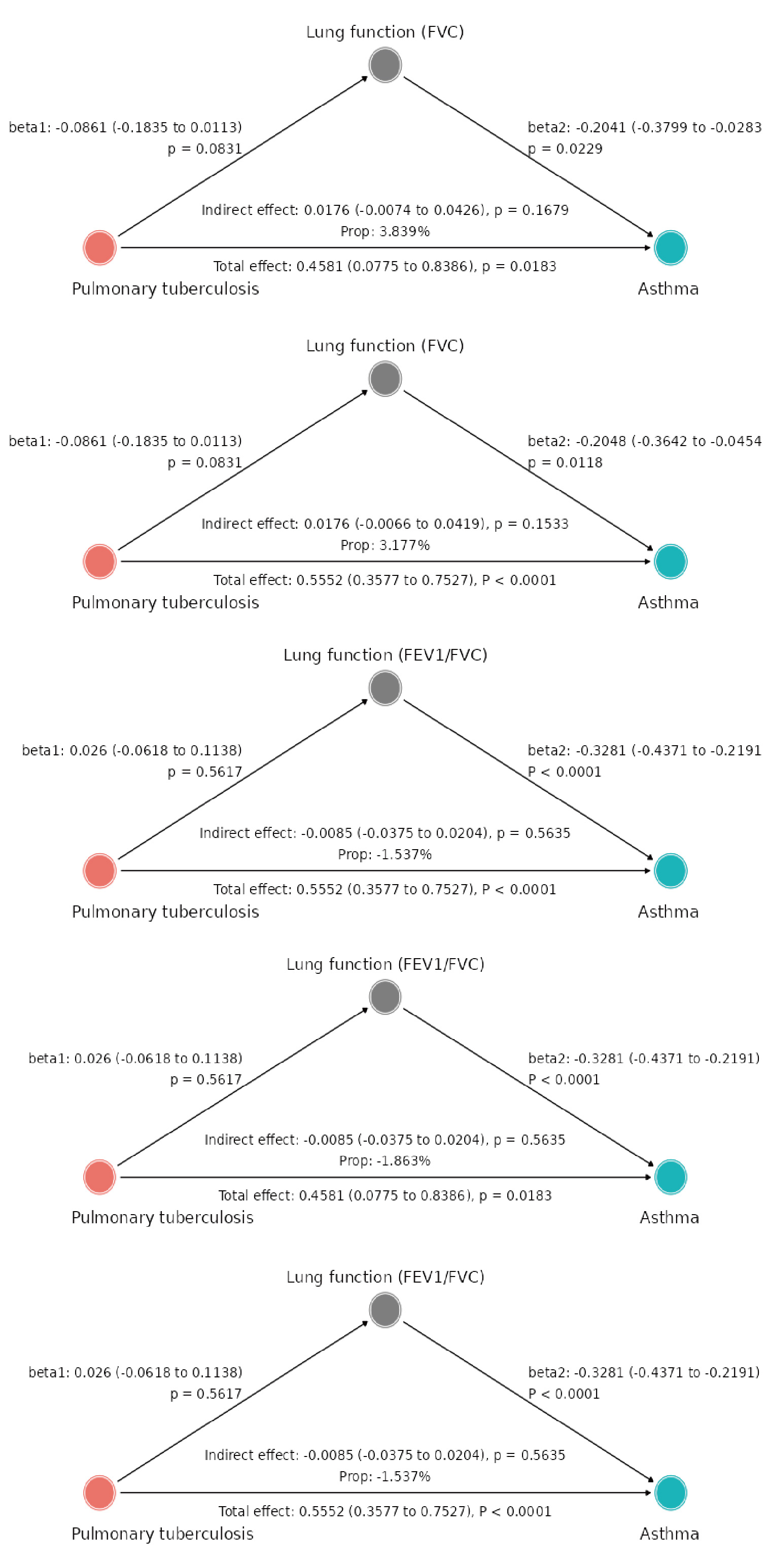

Supplement: Supplementary file 1 [file Image3.jpg]

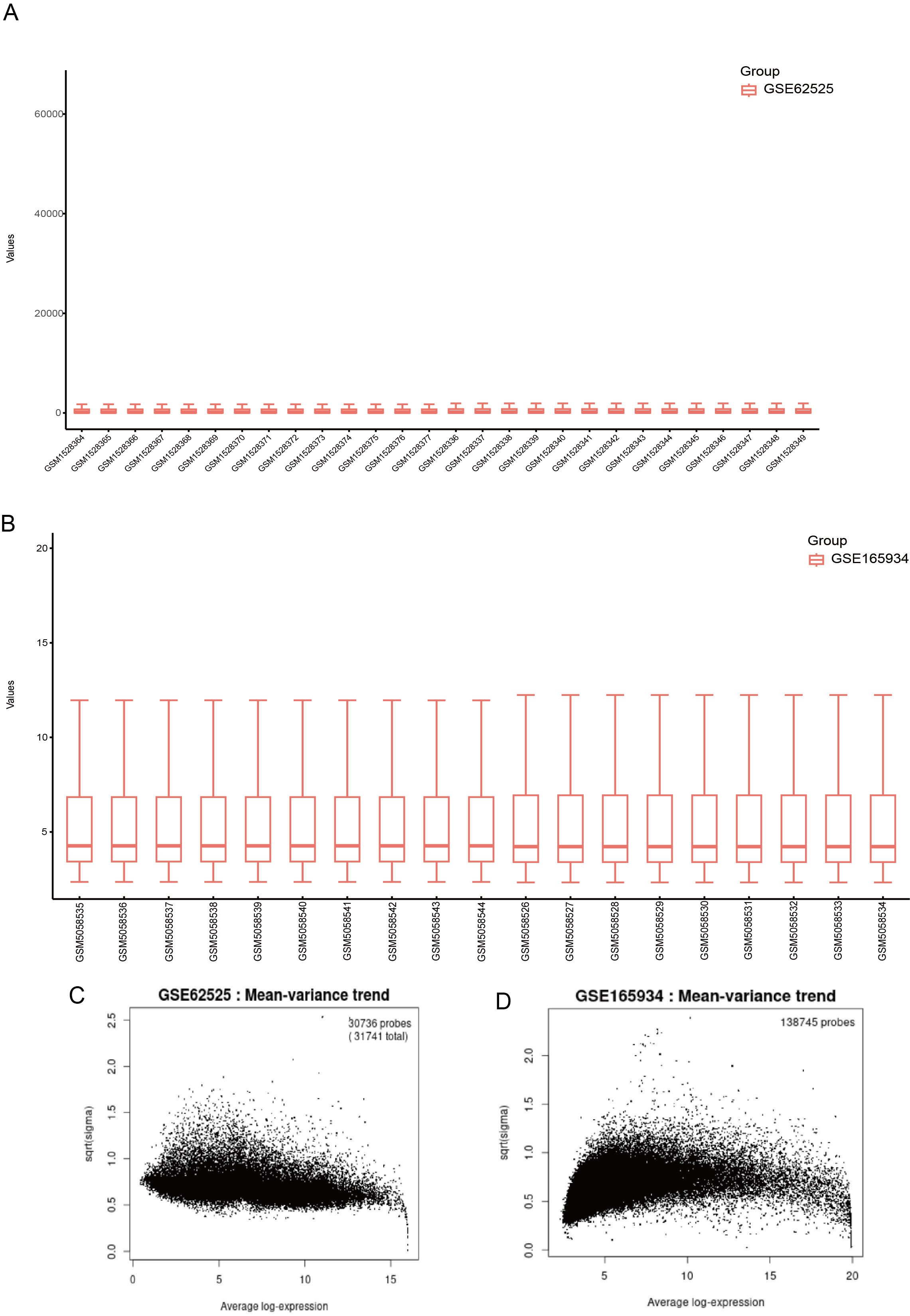

Supplement: Supplementary file 2 [file Image2.jpg]

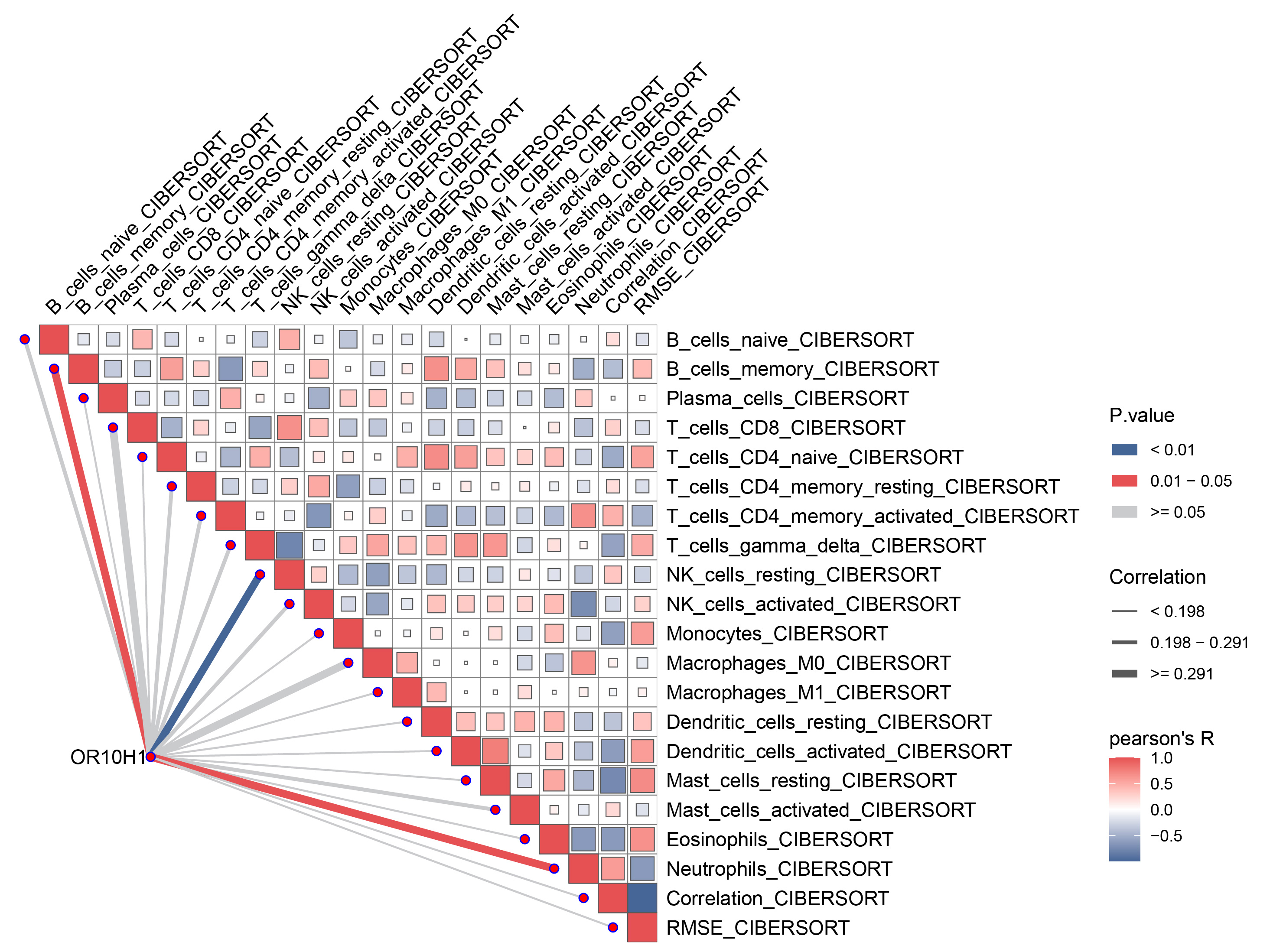

Supplement: Supplementary file 4 [file Image4.jpg]

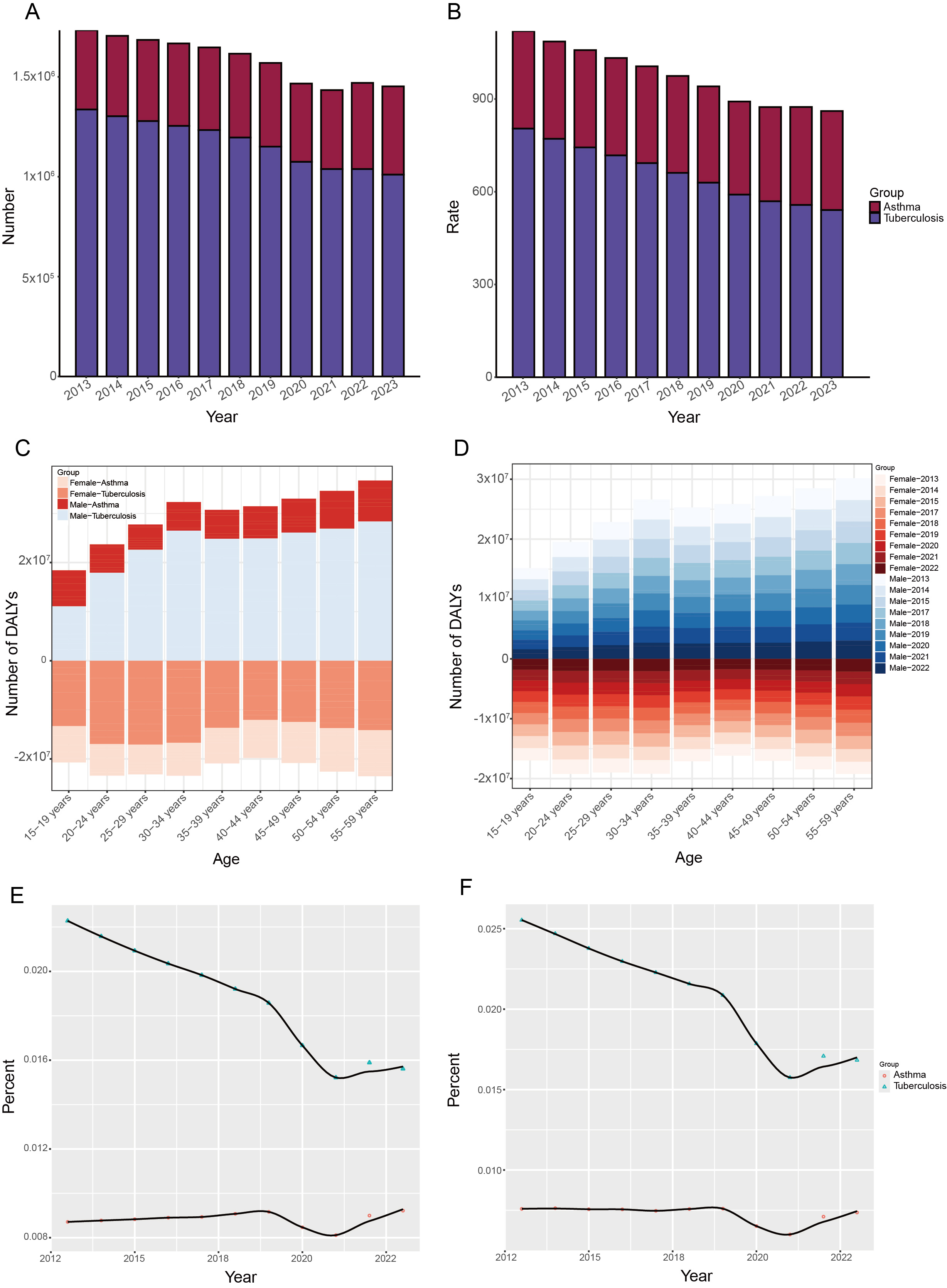

Supplement: Supplementary file 5 [file Image1.jpg]
